# Recreation in Relation to the Health of the People

**Published:** 1891-02-14

**Authors:** 


### Recreation in Relation to the Health of the People.

### III.—WORKING UP.

We need make no apology for returning to the Royal Victoria Hall and its spirited lady manager, for if it "began at the bottom," it has certainly "worked up," as its history will show.

As the story told in our last article represented the music-hall singer in his worst aspect, we hasten to state that Miss Cons speaks very warmly of these singers as a body. It is very generally not they, but the managers who engage them, and who pander to the worst tastes of those whom at the same time they brutalise with their vile decoctions, that are really to blame in the matter. A pathetic story was told by a young *artiste* who applied for an engagement at the Victoria Hall, and was asked if she liked appearing in the scanty costume depicted in her photograph. When the manager of another hall at last bullied her into appearing in that costume, she said, she only just managed to get across the stage and then fainted in the opposite wing. "But," she added (is not this the most pathetic touch of all?), "I'm used to it now, and I don't mind it."

We are glad to add, however, that of late there has been a considerable improvement in music-hall entertainments. Part of this improvement may be traced, we feel sure, to the example set by the new departure made by Miss Cons and other devoted workers; and we may quote in this connection a remark made in the report of the Victoria Hall with reference to the ballad concerts, which are now one of its most important features: "Competition in this branch has enormously increased of late. If imitation is the highest flattery, the promoters of the concerts ought to feel much flattered, for it is said that there are now twenty-three such entertainments weekly in Lambeth." These are probably new rather than reformed entertainments; still, there can be no doubt that the example of the Victoria Hall has been a great help both to respectable managers and to singers.

But the general improvement in our music-halls is doubtless also owing to Mr. MacDougall and the County Council. Whatever may be thought of the wisdom of all their proceedings, it is indisputable that their action has greatly altered the character of music-hall performances, and that many persons having large experience in the matter speak of it with deep gratitude. "Hurrah for MacDougall, say I!" one music-hall singer exclaims. "The respectable artistes can't be shopped [engaged] fast enough now, there is such a demand for them."

We may mention that regular variety entertainments are now given at the "Old Vic." on Saturday nights only. On Tuesdays "Penny Science Lectures" attract good audiences; on Thursdays there are popular ballad concerts, selections from various popular operas, illustrated by tableaux, being often given; Friday is the temperance night. The Morley Memorial College, which has lately been opened in connection with the hall, will be spoken of later on. Everything is in excellent working order, and the hall and college are well worth a visit, and (shall we add?) a little more help also; for until Parliament permits the Charity Commissioners to give their promised endowment to the hall (and in these days of obstruction, who can predict when that will be?), there is a great and a pressing need for funds.

We have described the work of this hall at some length, because it affords, as has been said, an excellent instance of the success attending efforts to "begin from the bottom and work up," and also because we think the hall is in some danger of being lost to view, owing to the superior size of its younger sister, the People's Palace. Not that they can ever be rivals, for each has its own separate work to do, one on a large scale in East London, the other, on a smaller scale, in its own south-eastern district: but it is only fair to remember that years before a stone of the People's Palace had been laid—probably before the "Palace of Delight" had reared its stately piles in its author's imagination—the "Old Vic." was steadily at work, slowly but surely making good its footing on what was then untried and even dangerous ground.

If variety entertainments are the very thinnest end of the wedge, shows of various kinds run them very near. One needs but very little culture, if any, to appreciate exhibitions of poultry, donkeys and ponies, cats, dogs, and rabbits; and Sir Edmund Currie shows that he knows how to cater for the East-end public, and to make the People's Palace popular among them, by the care which he takes to provide these shows and to make them attractive. From cat and donkey to flower shows, and exhibitions of various kinds of work, is a step upwards, and from them to picture exhibitions, perhaps another; but all these have proved very attractive at the People's Palace, while the success of Mr. Barnett's Bethnal Green Picture Exhibition is too well known to need mention here.

With regard to music, no doubt a ballad concert is most warmly and immediately appreciated by an uncultured London audience; indeed, it "takes" at once, and continues —as a good ballad concert deserves to do—to draw; but it is by no means a difficult task to lead up through these ballad concerts to performances of more difficult music. Some there already are, even among these very uncultured classes, who can appreciate it; for, as Miss Cons says, "it can never be too often insisted on that the working-classes are no more cast in one mould, and possessed of identical tastes in their amusements, than any other class"; and others can easily be educated up to it. The Popular Musical Union reports that "the taste of the people of the East and South of London is improving, so that works of the great masters need no longer, as in years gone by, the additional attraction of a military band to make them acceptable; but large and appreciative audiences now assemble at Mile End, Whitechapel, and Bermondsey, to listen to and enjoy 'Judas Maccabæus,' the 'Creation,' and the 'Redemption.'"

This society has, indeed, substituted for its old name—the "Popular Ballad Concert Committee"—the more comprehensive one of the "Popular Musical Union"—surely a significant change; and both it and the Kyrle Society do excellent work in supplying good concerts in various parts of London. Both societies appeal for more help, one needing good voices for the strengthening of its choir; the other, assistance in its orchestra. Why do not more ladies and gentlemen volunteer to help in the twofold work of providing recreation for the working classes, and of raising and purifying their tastes?

That it *should* be a twofold work we are anxious to insist. It is easy enough to amuse the people by coming down to their level, and indulging in the inanities—and, we fear we must add, the vulgarities—of the ordinary music hall type of song, though perhaps the amusement which it occasions might hardly be relished by us, if we learnt all its component parts; but surely cultivated people might aim at something higher than this. We feel great sympathy for the East End clergyman who, not long ago, invited some ladies of title to help to amuse a working-class audience, and who heard with shame songs sung by these ladies which would at once have been declined had they been submitted to him by his East End helpers.

It is said—with how much truth we know not—that this music-hall type of song appeals very strongly to a certain portion of our youthful aristocracy. Well, if so, let them keep these songs for their own firesides, and their own privileged circles, as working-men do their everyday clothes; and when they come out to meet the people, let them provide a better style of entertainment, which at least they can understand, if they do not value—or else let them stay at home. For the classes and the masses to draw more closely together is an excellent thing; but if they are only to hob-nob over amusements such as these, with the aristocrat taking the lead, we had better keep him away from the working man, trusting (that is all that is left to us) that distance may still "lend enchantment to the view."

